# Metabolic Profiling of *Heliotropium crispum* Aerial Parts Using HPLC and FTIR and In Vivo Evaluation of Its Anti-Ulcer Activity Using an Ethanol Induced Acute Gastric Ulcer Model

**DOI:** 10.3390/metabo12080750

**Published:** 2022-08-16

**Authors:** Syeda Farheen Fatima, Saiqa Ishtiaq, Manar O. Lashkar, Fadia S. Youssef, Mohamed L. Ashour, Sameh S. Elhady

**Affiliations:** 1 Punjab University College of Pharmacy, University of the Punjab, Lahore 54000, Pakistan; 2 Department of Pharmacy Practice, Faculty of Pharmacy, King Abdulaziz University, Jeddah 21589, Saudi Arabia; 3 Department of Pharmacognosy, Faculty of Pharmacy, Ain-Shams University, Abbasia, Cairo 11566, Egypt; 4 Pharmacy Program, Department of Pharmaceutical Sciences, Batterjee Medical College, Jeddah 21442, Saudi Arabia; 5 Department of Natural Products, Faculty of Pharmacy, King Abdulaziz University, Jeddah 21589, Saudi Arabia

**Keywords:** gastric ulcer, ethanol, omeprazole, flavonoids, *H. crispum*, drug discovery, health care

## Abstract

This study explored the antiulcer potential of methanol extract and fractions of *Heliotropium crispum* roots against the ethanol-induced gastric ulcer model in rats. Metabolic profiling of *H. crispum* aerial parts using Fourier-transform infrared spectroscopy (FTIR) revealed the presence of different metabolites with various functional groups. Meanwhile, High Performance Liquid Chromatography (HPLC) revealed the presence of three main peaks assigned to myricetin, quercetin, and kaempferol. In vivo, antiulcer activity results showed that the disease control group displayed five tiny ulcers less than 2 mm in diameter in addition to two hemorrhagic streaks. However, in the standard control group, only one small ulcer was visible for the total methanol extract. Gastric tissues and contents were evaluated to determine many parameters such as ulcer score, ulcer index, percentage inhibition of ulcer, gastric pH, gastric juice volume, and acidity. Results were endorsed by histopathological evaluation; gastric pH and mucus content were significantly increased, but gastric juice volume was significantly decreased. All fractions showed a significant decrease in ulcer index and % inhibition except the *n*-hexane fraction, whose results were insignificant compared to the disease control group. Thus, it was concluded that *H. crispum* shows an antiulcer effect by decreasing gastric juice volume and acidity, whereas gastric pH and mucus contents were increased that is attributed to the synergistic action of its detected polyphenolic compounds.

## 1. Introduction

Gastric ulcer constitutes one of the most hazardous gastrointestinal tract disorders widely spread all over the globe, where 4–10% of the world population suffer from this disease [1]. It is mainly attributed to the imbalance between injurious and mucosal defense factors [2,3]. Mucosal damage or injury due to harmful factors like acids, pepsin, bacteria (*Helicobacter pylori*), non-steroidal anti-inflammatory drugs (NSAIDs) abuse, smoking, stress as well as ingestion of alcohol will ultimately result in the development of gastric ulcers [4]. It is noteworthy to highlight that alcohol has been proved to be one of the major reasons that trigger gastric mucosal damage. Alcohol ingestion results in delayed stomach emptying time (gastroparesis), decreased intestinal motility, and increased wall permeability, finally leading to ulcer development [5]. This pathological condition is characterized by severe pain in the abdomen and heartburn, in addition to various gastrointestinal tract (GIT) complications [6]. Although ulcer is not considered a deadly disease, it can result in complications like GIT bleeding, GIT perforations, and ulcer pricking into adjacent organs that lead to death [7].

The treatment regimen includes either aggressive neutralizing factors or stimulating defense factors like mucus, bicarbonate, prostaglandin, and antioxidants [8]. The main goal of the treatment strategy is to relieve the associated pain in addition to the healing ulcer and preventing ulcer recurrence [9]. Current antiulcer medications have numerous adverse effects manifested by proton pump inhibitors that cause severe calcium, vitamin B12, and iron depletion in the body owing to malabsorption [10]. Furthermore, H_2_ receptor antagonists may cause gynecomastia and impotence [11].

Thus, there is a great need to discover less aggressive and more diversified bioactive compounds of natural origin that show higher safety, fewer adverse effects, cheaper prices that are more welcomed by many patients compared to synthetic drugs [12]. Plants have been everlasting sources of treatment for various ailments and diseases for centuries [13]. Many studies have proven the gastro-protective effects of many plant extracts based on the reported activity in traditional medicine [14]. The significant biological activities of plant extracts are mainly attributed to the constituting bioactive secondary metabolites [15].

Genus *Heliotropium* belongs to family Boraginaceae, which includes nearly 250 species that are distributed among the world; meanwhile, only about twenty-three species are growing in various areas in Pakistan. The word “heliotrope” described the property of turning the leaves of the plant in the direction of the sun [16]. *H. crispum* is an important member of this family, which is traditionally used to treat many diseases like kidney problems, skin problems as well as urinary tract infections [17,18]. Recently, its methanol extract revealed high levels of total phenolic content in addition to identifying several individual phenolic compounds with potent antioxidant abilities and enzyme inhibitory potential. Reversed phase ultra-high-performance liquid chromatography coupled to electrospray ionization quadrupole-time-of-flight mass spectrometry (RP-UHPLCQTOF-MS) metabolic profiling revealed the existence of important pyrrolizidine alkaloids, peptides, iridoids, and phenolic compounds, which are popular for their antioxidant activities [16].

Tracing the current literature nothing was found regarding the antiulcer potential of the plant. Thus, herein, we aimed to investigate the antiulcer activity of *H. crispum* in vivo against ethanol induced gastric ulcer models in rats. This was accompanied by various biochemical and histopathological examinations to ascertain the obtained results. Additionally, metabolic profiling of *H. crispum* total methanol extract using FTIR (Fourier-transform infrared) spectroscopy and High Performance Liquid Chromatography (HPLC) was also performed.

## 2. Results

### 2.1. Metabolic Profiling of H. crispum Aerial Parts

#### 2.1.1. Fourier-Transform Infrared Spectroscopy (FTIR)

Metabolic profiling of *H. crispum* aerial parts using Fourier-transform infrared spectroscopy (FTIR) revealed the presence of different metabolites evidenced by the presence of different peaks assigned to various functional groups. Results of the IR spectrum illustrated in Table 1 showed the presence of stretching vibrations that appear at 3296.5 cm^−1^ and accounted for the bonded O-H bands of carboxylic acid [19] as well as primary amides, secondary amides as well as polymer alcohols and phenols. Meanwhile, the peak at 2918.0 cm^−1^ could be assigned to the presence of hydroxyl group and secondary free or bonded NH strand. Furthermore, the fingerprint region accounts for the presence of various functional groups as illustrated in Table 1. The FTIR spectrum of *Heliotropium crispum* aerial parts powder was illustrated in Supplementary Material Figure S1.

#### 2.1.2. High Performance Liquid Chromatography (HPLC)

**Figure 1:**
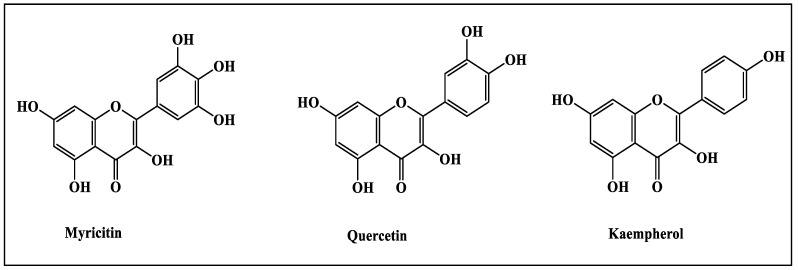
Compounds identified in *H. crispum* total methanol extract (CME) as well as its various fractions using HPLC.

Metabolic profiling of *H. crispum* aerial parts using High Performance Liquid Chromatography (HPLC) revealed the presence of three main peaks assigned to myricetin, quercetin and kaempferol appearing in the different fractions that are identified based upon the injection of standards as illustrated in Table 2 and Figure 1. HPLC chromatograms of the different extracts of *Heliotropium crispum* aerial parts were illustrated in Figure S2.

### 2.2. In Vivo Determination of H. crispum Anti-Ulcer Activity Using Ethanol-Induced Acute Gastric Ulcer Model

#### 2.2.1. Macroscopic Analysis of Gastric Mucosa

**Figure 2:**
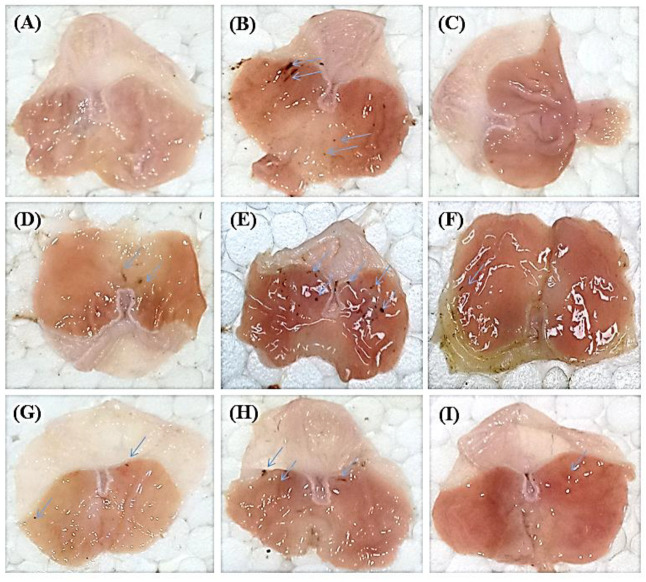
Macroscopic view of stomach mucosa in the acute gastric ulcer model in the *H. crispum* total methanol extract (CME) treated gastric tissues as well as its various fractions; normal group showing no ulcer(**A**), disease control group showing 5 small ulcers and 2 hemorrhagic streaks(**B**), omeprazole treated group showing one small ulcer(**C**), CME treated group showed only 1 small ulcer (**D**), *n*-hexane treated group showing many small ulcers (**E**), chloroform treated group showing one ulcer and one hemorrhagic streak, (**F**), ethyl acetate treated group showing 2 small ulcers and (**G**), *n*-butanol treated stomach showing 2 small ulcers and 2 large streaks (**H**) and remaining aqueous fraction (**I**) treated groups showing red coloration and small ulcers. Blue arrows show ulcers and hemorrhagic streaks produced due to ethanol-induced damage to stomach mucosa.

The gastric wall of each rat was examined by naked eye and then by the hand lens. A macroscopic view of stomach walls of normal group showed no ulcer or streaks in contrast to the disease control group that displayed five small ulcers less than 2 mm in diameter in addition to two hemorrhagic streaks. Meanwhile, in the standard control group, only one small ulcer was visible and similarly for the total methanol extract that displayed only one small ulcer. However, the *n*-hexane treated stomach showed many small ulcers less than 2 mm in size, whereas, in chloroform treated stomach, one ulcer greater than 2 mm and one hemorrhagic streak were visible. For the ethyl acetate fraction, two small ulcers less than 2 mm were detected, whereas two small ulcers less than 2 mm and two large streaks were observed in the *n*-butanol treated stomach; however, red coloration and small ulcers less than 2 mm were observed in the remaining aqueous fraction treated gastric tissues (Figure 2).

#### 2.2.2. Determination of Ulcer Score, Ulcer Index and Percentage of Ulcer Protection

The antiulcer potential of any drug can be tested by using ulcer index which constitutes a measure of gastric mucosal lesion. Gastric damage was measured by mucosal lesions and spots of different sizes. The incidence of ulcer occurrence, ulcer score, ulcer index (UI) and % ulcer inhibition of experimental as well as control groups in acute gastric ulcers are shown in Table 3. UI for the ethanol treated group was 125.17 with 100% incidence of ulcer occurrence. Meanwhile, treatment with omeprazole, CME and the different fractions considerably reduced the UI particularly for CME, chloroform and ethyl acetate fractions estimated by 17.33, 17 and 17, respectively showing 84.41%, 86.42% and 86.42% protection versus ulcer occurrence. It is worth mentioning that CME, chloroform and ethyl acetate fractions showed ulcer protection approaching that of omeprazole that showed UI of 17 with 84.41%protection. However, *n*-hexane, *n*-butanol and remaining aqueous fraction showed moderate anti-ulcer activity with UI estimated by 69.33, 86.17 and 68.50, respectively with 44.60%, 31.03% and 45.27% protection, respectively.

#### 2.2.3. Determination of Gastric Juice Volume, pH and Total Acidity

**Figure 3:**
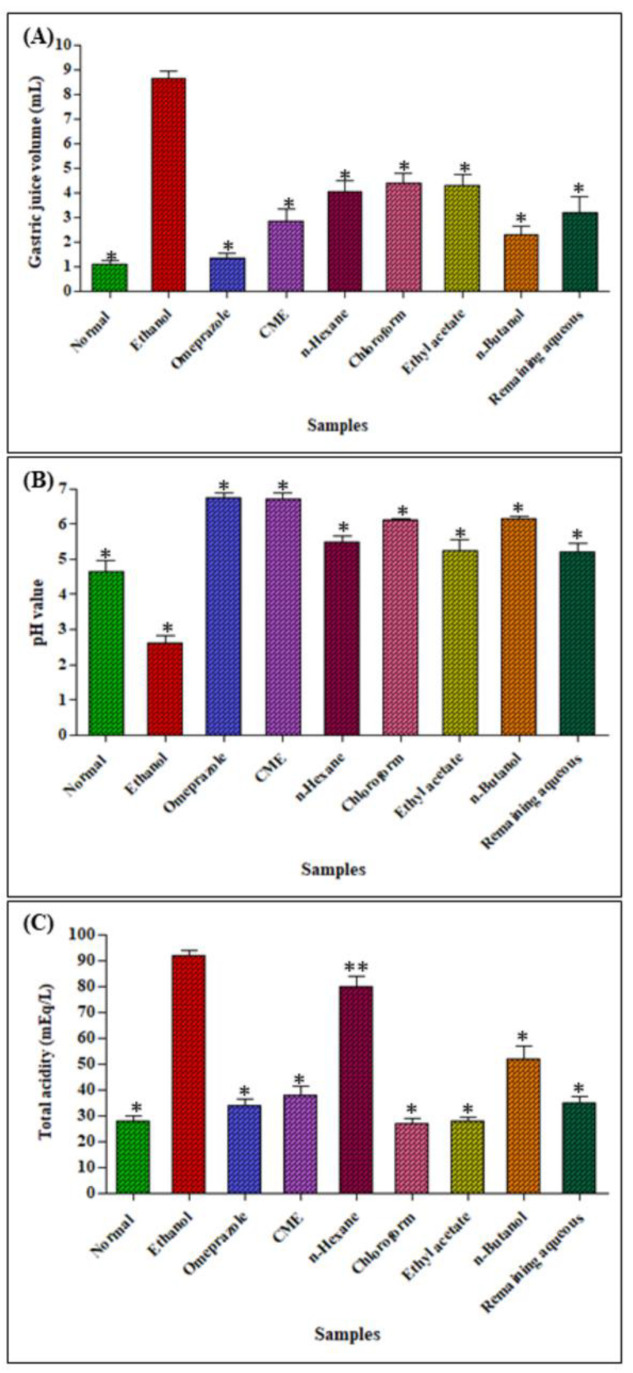
Effect of *H. crispum* total methanol extract (CME) as well as its various fractions on gastric juice volume (**A**), pH (**B**) and total acidity (**C**) in ethanol induced acute ulcer; the results are expressed in the form of Mean ± SEM. Significant at *p* < 0.01 * and 0.5 ** from the diseased control group.

Regarding the properties of the gastric juice of ulcer control group (Figure 3), it showed greater gastric juice volume, lower pH as well as high total acidity when compared to a normal control group. A significant increase in pH (*p* < 0.001) with a concomitant decrease in gastric juice volume and total acidity in ulcer induced rats was observed upon pretreatment with omeprazole, CME, chloroform and *n*-butanol fractions. There was a significant increase in pH (*p* < 0.001) of CME, *n*-butanol and remaining aqueous fraction when compared with group disease control animals. The omeprazole pretreated group also showed a significant increase in pH when compared with the disease control animals. Furthermore, CME, *n*-hexane, chloroform and *n*-butanol pretreated groups showed a decrease in total acidity (*p* < 0.001) when compared with a disease control group. The standard treated group also showed a decrease in total acidity when compared to the disease control group. Concerning the gastric juice volume, omeprazole and *n*-butanol gave comparable results with the normal control group.

#### 2.2.4. Effect on Total Protein and Total Mucus Content

The alcian blue binding capacity was utilized as a marker for the quantitative determination of gastric mucus content in the stomach. Treatment with ethanol caused a marked reduction total gastric mucus content estimated by 15.59% compared to the normal control group with concomitant reduction in alcian blue binding capacity. Pretreatment with omeprazole as well as CME, *n*-hexane, chloroform, ethyl acetate, *n*-butanol and remaining aqueous fractions caused a pronounced amelioration in gastric mucus content approaching that of the normal control group causing elevation in gastric mucus content by 12.40, 12.40, 3.84, 16.33, 7.30, 17.64 and 12.05%, respectively when compared to the diseased group. Concerning the total protein content, treatment with ethanol resulted in a significant decline in total protein content estimated by 189.29% compared to the normal control group. On the contrary, pretreatment with omeprazole as well as CME, *n*-hexane, chloroform, ethyl acetate, *n*-butanol and remaining aqueous fractions caused a marked elevation in total protein content estimated by 142.92, 178.18, 79.11, 182.47, 162.88, 122.20 and 106.54%, respectively, with respect to the diseased group (Table 4).

#### 2.2.5. Histological Examination of Stomach Wall

**Figure 4:**
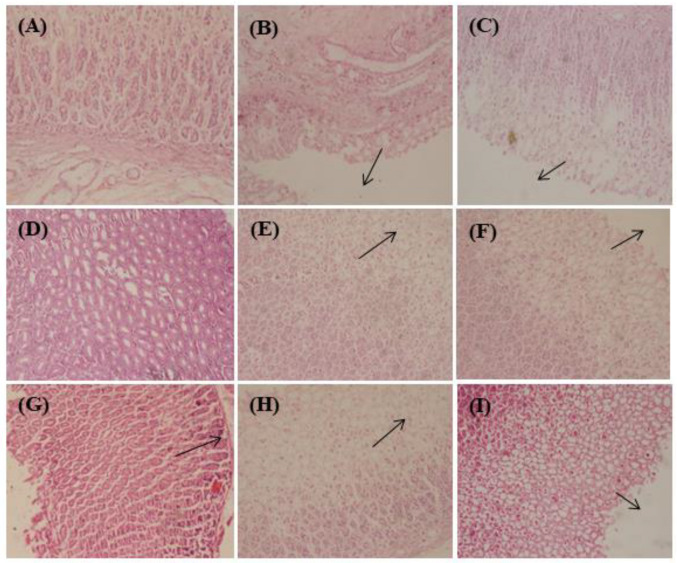
Histopathological examination of stomach mucosa in the acute gastric ulcer model in *H. crispum* total methanol extract (CME) treated gastric tissues as well as its various fractions; normal group (**A**), disease control (**B**), omeprazole (**C**), CME (**D**), *n*-hexane (**E**), chloroform (**F**), ethyl acetate (**G**), *n*-butanol (**H**) and remaining aqueous fraction (**I**) treated groups. Black arrows indicate damage to peptic epithelium.

Histopathological assessment of gastric mucosa illustrated in Figure 4 showed extensive damage to gastric mucosa triggered by ethanol consumption that extended deeply with concomitant mucosal and sub-mucosal edema and leukocytes infiltration. In contrast, omeprazole, CME, *n*-hexane, chloroform, ethyl acetate and *n*-butanol pretreatment displayed a pronounced protection to stomach mucosa accompanied by a notable decline in edema and leukocytes infiltration of sub-mucosal layers; meanwhile, the remaining aqueous fraction treated group showed necrosis and cellular degeneration. From Figure 4, it is clearly obvious that the disease control group showed severe damage to peptic epithelium; the omeprazole treated group revealed mild damage to the gastric epithelium; CME treated group exhibited normal gastric tissue, whereas the *n*-hexane treated group showed disorganized gastric epithelium; meanwhile, chloroform, ethyl acetate and *n*-butanol treated groups showed less damage to peptic epithelium.

## 3. Discussion

Ulcer or gastric mucosal layer damage is usually controlled by a combination of factors [20]. The most important factor is the balance between the aggressive factors represented by gastric juice comprising hydrochloric acid and pepsin and the protective factors like mucus and bicarbonate secretion. Additionally, prostaglandins, mucosal blood flow and nitric oxide are also important factors which play a vital role in maintaining the integrity of gastric mucosa [21]. There is a compulsive need to develop dosage regimens from natural sources which are known to have less side effects as compared to proton pumps and H_2_-receptor antagonist [22].

Basically, alcoholism is a proved source to produce different physiological disorders in bodies such as nervous, circulatory and endocrine system disorders [23].The absorption of ethanol is maximum from upper GIT so this area shows a maximum detrimental effect [24]. Ethanol produces gastric ulcers by causing turbulence in gastric secretions in addition to altering the gastric membrane permeability with subsequent production of free radicals. Ethanol causes damage to gastric mucosal lining, resulting in gastric tissue hemorrhage and necrosis. Ethanol perforates deeply and quickly in the gastric mucus secretion lining, which causes plasma membrane damage resulting in cell death due to enhanced accessibility of sodium and water in intracellular membrane accompanied by an enormous increase in intracellular calcium [25]. Gastric mucus secretion lining damage is related to lipid peroxidation and free radicals generation [26]. Alcohol induced ulcers in rats are blackish lesions varying in sizes and are parallel to greater curvature; meanwhile, they can be inhibited by various drugs like omeprazole [27]. The mucus layer in gastric tissue is mainly composed of mucin, proteoglycans, electrolytes, serum proteins and water. The main function of the mucus layer is to provide a physical barrier against gastric acids and exogenous irritants [21,28].

The gastro-protective effect of *H. crispum* was evaluated by using ethanol as an ulcerogenic agent due to its potential to cause damage to gastric mucosa. The current study showed that pre-treatment with CME, chloroform, ethyl acetate; *n*-butanol as well as remaining aqueous fractions caused a significant reduction (*p* < 0.0001) in ulcers causing a marked elevation in the mucus layer when compared to the ulcerated control group approaching that of omeprazole that was further confirmed by the histopathological examination.

Plant based products showed an antiulcer effect via effective cytoprotective action. Moreover, the increase in gastric mucus content also played a vital role in a cytoprotective mechanism. Furthermore, reduction in acidity also helps in an antiulcer effect due to a neutralization effect of gastric acids. Increase in mucus production is also a key factor to increasing the defense mechanism of stomachs against different deteriorating factors, which results in decreased incidence of ulcer production [29].

Additionally, many studies have correlated the protective effect versus gastric ulcer to the richness of the natural herbs with flavonoids and phenolic acids. The gastro-protective and anti-ulcer potential of the flavonoids identified in *H. crispum* was previously confirmed in a plethora of research in vitro, in vivo and in silico [30]. Quercetin represents one of the highly potent flavonoids associated with gastro-protection. It revealed a pronounced protective effect to gastrointestinal mucosa from acute lesions triggered by many in vivo induced ulcer models and versus diverse necrotic factors, comprising pylorus-ligation, restraint stress as well as many drugs as aspirin, reserpine, indomethacin and ethanol-induced gastric ulcers. Quercetin showed a strong prohibition versus *H. pylori* growth together with its pronounced antioxidant potential that significantly decreased the hazardous effect of ethanol-induced ulcers via prohibition of lipid peroxidation with concomitant stimulation of antioxidant glutathione peroxidase and superoxide dismutase activity as well [31,32]. In addition, at a dose of 50 and 100 mg/kg of oral administration of quercetin in the rat model, it effectively prohibited HCl plus ethanol-induced gastric mucosal injury with concomitant reduction of thiobarbituric acid-reactive substances content in the injured mucosa in rats [33]. It also inhibited gastric proton pump and lipoxygenase pathway and elevated neutral glycoproteins content in the gastric mucosa. Thus, it elicited a recovery of the defensive capacity versus absolute ethanol aggressive effect with concomitant elevation of prostaglandin synthesis postulating its role in increasing the amount of mucus and its incorporation in ulcer prevention [34]. Furthermore, in silico studies showed that quercetin exerted a notable binding affinity score of −6.418 kcal/mol with the active site of the gastrin proton pump (PDB code 5YLU). It forms one hydrogen bond with Glu 343 similar to the co-crystallized ligand and two additional hydrogen bond interactions with Ala 335 and Asn 792 amino acid residues. It also showed a binding affinity of −6.013 kcal/mol with the active site of M3 receptor (5ZHP) forming with three hydrogen bonds with the amino acid residues of Ala 238, Cys 532 and Tyr 529, in addition to hydrophobic interaction with Tyr 506 [35].

Moreover, myricetin showed potent antiulcer potential in many previous studies in vitro, in vivo and in silico owing to the presence of various phenolic hydroxyl groups [36]. In vitro, it prohibited H^+^, K^+^-ATPase with a sub-micromolar IC_50_ value in an enzyme-based assay performed by freeze-dried tubulovesicles obtained from a hog stomach. It inhibited both Na^+^, K^+^-ATPase and Ca^2+^-ATPase as well in a dose-dependent manner which basically depends on the existence of phenolic hydroxyl groups [37]. Meanwhile, in vivo, at a dose of 12 mg/kg, it significantly reduced alcohol-induced gastric ulcer, hemorrhage, hyperemia and epithelial cell loss in the gastric mucosa. In addition, it decreased malondialdehyde (MDA) level with concomitant elevation in total glutathione (GSSG/GSH) and superoxide dismutase (SOD) in gastric tissues. Additionally, it increased cyclooxygenase-1 (COX-1) and prostaglandin E_2_ (PGE_2_) expression and reduced nuclear factor kappa B (NF-*κ*B) phosphorylation and thus accounting for its anti-ulcer potential [38]. Moreover, in silico studies showed that inhibited the gastric proton pump ameliorating gastric acid secretion evidenced by its firm binding with the gastrin proton pump (5YLV) with binding free energies of −18.30 kcal/mol forming two conventional H-bonds with Glu 900 and Gln 127 [39].

Regarding kaempferol, it showed significant anti-ulcer activity in vivo in acute ethanol-induced lesions to the gastric mucosa in mice when orally administered at the dosses of 40, 80 or 160 mg/kg. It reduced ulcer index, elevated the preventive index, completely shielded the mucosa from lesions and protected gastric mucosal glycoprotein. Additionally, it reduced MPO as well as the levels of pro-inflammatory cytokine represented by TNF-α, and IL-1β and enhanced NO levels [40,41]. In silico, kaempferol binds to M3 receptor (5ZHP) active site with binding to the active site of the M3 receptor with a binding affinity of −6.081 kcal/mol forming hydrogen bonding between its phenolic hydroxyl groups and Tyr 529 and Thr 231 amino acids residues at the binding site together with one hydrophobic interaction with Tyr 506 residue [35].

All these findings proved the antiulcer effect of CME, chloroform, *n*-butanol and aqueous fractions of *H. crispum* against ethanol induced gastric injury related with ethanol intake. Significant decreases were observed in both ulcer score and ulcer index and accompanied by a significant inhibition percentage of gastric ulcer.

## 4. Materials and Methods

### 4.1. Plant Material

Whole plant of *H. crispum* was collected from Cholistan desert, Punjab, Pakistan. It was identified by plant taxonomist Muhammad Abdullah, and a voucher specimen was deposited at the herbarium of the Cholistan Institute of Desert Studies, Islamia University of Bahawalpur, Pakistan under voucher number CIDS/ IUB-0502/23.

### 4.2. Drugs and Chemicals

Omeprazole was obtained from Zafa Pharmaceuticals (Karachi, Pakistan), whereas BSA (Bovine Serum Albumin) was purchased from Bioshop, (Burlington, ON, Canada). Other chemicals and solvents such as sodium carbonate, methanol, *n*-hexane, chloroform, ethyl acetate, *n*-butanol, sodium potassium tartarate, copper sulphate, Folin–Ciocalteu (FC) reagent, sodium acetate, magnesium chloride, alcian blue, glacial acetic acid, diethyl ether, normal saline and formalin were brought from Merck (Karachi, Pakistan), and they were all of analytical grade.

### 4.3. Preparation of H. crispum Methanol Extract and Successive Fractionation

Whole plants of *H. crispum* were washed with water to remove dust and sand particles, then dried in the shade and ground in coarse particles using a mechanical mill. In addition, 500 g of the plant powder were then soaked in methanol (2.5 L) for 14 days with occasional shaking. This extract was then filtered by using Whatsman No. 1 filter paper. Filtrate (crude methanol extract) was concentrated by using rotary evaporator at 40 °C, and then it was dried in a hot air oven. Dried crude methanol extract (CME, 30 g) was dissolved in distilled water and then subjected to successive fractionation using *n*-hexane, chloroform, ethyl acetate and *n*-butanol in an order of increasing polarity that result in formation of *n*-hexane (6.5 g), chloroform(9 g), ethyl acetate(8 g), *n*-butanol (2.5 g) and aqueous (4 g) fractions, respectively. These fractions were stored in an amber colored, air tight container in the refrigerator for further use.

### 4.4. Metabolic Profiling of H. crispum Aerial Parts

#### 4.4.1. Fourier-Transform Infrared Spectroscopy (FTIR)

Completely dried plant powder was used for FTIR analysis where the sample was loaded in FTIR spectrometer with a range of 400–4000 cm^−1^ as previously described by Ahmed et al. [42].

#### 4.4.2. High Performance Liquid Chromatography (HPLC)

Flavonols in above given samples were analyzed by using HPLC (model LC-10 A, Schimadzu, Kyoto, Japan), having 2 LC-10 AS pumps, SCL-10A system control unit, Rheodyne injector, CTO-10A column oven, SPD-10A UV-vis detector and data acquisition class LC-10 software, were used. A 20 µL filtered sample was injected in analytical Supelco (Supelco Inc., Supelco Park, Bellefonte, PA, USA) ODS reverse phase (C18) column (250 × 4.6 mm; 5 µm particle size). Two solvents’ systems A: contained 3% trifluoroacetic acid and B: contained acetonitirile and methanol (80:20 *v*/*v*) were used. The chromatographic separation was performed by isocratic elution of the mobile phase (mixture of solvent A and B, 50:50 *v*/*v*) that was filtered under vacuum through a 0.45 µm membrane before use) at a flow rate of 1.0 mL min^−1^ at 30 °C. Detection was performed at a wavelength of 360 nm. Flavonols (kaempferol, quercetin, myricetin) present in samples were identified by comparing with standards (sigma chemicals Co, St Louis, MO, USA) [43].

### 4.5. In Vivo Determination of H. crispum Anti-Ulcer Activity Using an Ethanol-Induced Acute Gastric Ulcer Model

#### 4.5.1. Experimental Animals

Wistar rats of either sex were used for this study ranging in weight from 120 to 150 g. Laboratory animals were provided by University College of Pharmacy, University of the Punjab, Lahore, Pakistan. Animals were acclimatized for one week in the animal house of the above-mentioned university. Animals were provided with standard pellet diet and tap water *ad libitum.* Guidelines for use of laboratory animals by bio-ethical committee from the University of the Punjab, Lahore, Pakistan were properly followed during this study under the ethical code (No. D/209/FIMS).

#### 4.5.2. Experimental Protocol

Ethanol induced ulcer model was used following the method previously described by Hollander et al. [44,45]. Animals were divided into nine groups with six rats in each group. The first group, normal control, was given distilled water orally. The second group, disease control, was given ethanol only at a dose of 5 mL/Kg body weight; however, the third group, the standard group, was orally administered the standard drug omeprazole at a dose of 20 mg/Kg. From the fourth to the ninth groups, animals were orally administered with crude methanol extract, *n*-hexane, chloroform, ethyl acetate, *n*-butanol and aqueous fractions at a dose of 1000 mg/kg body weight, respectively. All the experimental animals were fasted for 24 h before the beginning of study but provided with water *ad libitum*. All groups excluding the normal control and the disease control groups were provided with respective doses. After 30 min of dosing, all experimental groups except the normal control group were given ethanol at a dose of 5 mL/Kg body weight. After 30 min, all animals were sacrificed and stomachs were removed. Stomach contents were collected in centrifuge tubes. Stomachs were cut along the greater curvature and washed with ice cold saline. Stomach tissues were observed for ulcer scores after placing on a soft white board. The tissues were preserved in formalin for hematological examination. Ulcer index, incidence of ulcer, percentage of ulcer inhibition, gastric pH, gastric volume, gastric acidity, mucous content as well as protein content were calculated both in gastric tissue and gastric juice [1].

#### 4.5.3. Gross and Histological Evaluation of Stomach Tissues

Removed stomach was washed properly with normal saline to remove all traces of gastric content and blood clots. The inner side of stomach was observed for lesions present on the inner side of stomach with the use of 10X magnifying lens and dissecting microscope (CX 32, Olympus, Tokyo, Japan). The number of ulcers was counted by using these instruments.

##### Determination of Ulcer Scoring

Scoring of ulcers was done following the method previously reported by Kulkarni [45], where 0 = normal stomach, 0.5 = reddish stomach, 1 = red spot ulcer, 1.5 = hemorrhagic streak, 2 = deep ulcers, and 3 = perforations.

##### Determination of Ulcer Index

Lesions of every stomach were calculated and used for ulcer index calculation. It is calculated by using the following formula previously reported by Adane et al. [44]. Ulcer index = UN + UP + US × 10^−1^, where UN = average number of ulcers per animal; UP = % of animals with ulcers; and US = average number of severity scores.

##### Determination of Percentage of Ulcer Protection

Then, the percentage of protection was calculated by using following formula previously reported by Sharma et al. [46]:

$$\mathrm{Ulcer}\;\mathrm{index}=\frac{\mathrm{ulcer}\;\mathrm{index}\;(\mathrm{control})-\mathrm{ulcer}\;\mathrm{index}\;(\mathrm{treated})}{\mathrm{ulcer}\;\mathrm{index}\;(\mathrm{control})}\times 100$$

#### 4.5.4. Determination of Gastric Juice Parameters

After removal of stomachs, gastric contents were collected in graduated falcon tubes (10 mL) and were centrifuged at 3000 rpm for 10 min. The supernatant layer was checked for gastric juice volume and pH. Gastric pH was measured by using pH paper. Determination of gastric juice is an important parameter to check anti-secretory potential of plant extract [47].

##### Estimation of Total Acidity

In addition, 2–3 drops of phenolphthalein were added to 9 mL distilled water and 1 mL gastric juice. Titration was performed until a persistent red tinge appeared. Total acidity expressed in terms of mEq/L was calculated by using the following formula previously reported by Nwinyi et al. [47]:

(1) $$\mathrm{Acidity}=\frac{\mathrm{volume}\;\mathrm{of}\;\mathrm{NaOH}\times \mathrm{Normality}\;\mathrm{of}\;\mathrm{NaOH}}{0.1}\times 100$$

#### 4.5.5. Determination of Gastric Mucous

Glandular portions of excised gastric tissues were transferred to 0.1% *w*/*v* alcian blue solution (solution was made in 0.16 M sucrose solution with 0.05 M sodium acetate buffered solution, pH = 5). Mucous was rinsed twice with 250 mM sucrose solution after 15 minutes and 45 minutes. A mucus-dye complex was extracted with 10 mL of 500 mM of magnesium chloride solution for 2 h with continuous shaking for one minute. Four milliliters of the resulting solution were mixed with an equal volume of diethyl ether and centrifuged at 3000 rpm for 10 min. Absorbance was measured at 580 nm and a standard curve of alcian blue was plotted ranging from 20 µg/10 mL to 200 µg/10 mL, and it was used for mucus calculation where the results were expressed in µg of alcian blue extracted /g tissue [48,49].

#### 4.5.6. Histopathological Examination of the Gastric Tissues

At the end of study, stomach tissue was cut and rinsed with cold saline buffer and preserved with 10% formalin solution. In addition, 5 µm thick sections were cut with the help of microtome. Eosin and hemotoxylin stains were used for staining purposes. Slides were prepared and observed under light microscopes [50].

#### 4.5.7. Statistical Analysis

Results were expressed as mean ± SEM where data were analyzed by Student’s *t*-test with multiple comparisons with the groups. *p*-value (0.05) was considered to be statistically significant.

## 5. Conclusions

The current study revealed the antiulcer potential of the methanol extract of *Heliotropium crispum* roots and its fractions. This effect can be related to its anti-secretory potential, increased mucosal protection, anti-inflammatory and free radical scavenging activity of the plant. This is mainly attributed to the richness of plants with flavonoids as revealed from the High Performance Liquid Chromatography metabolic profiling, particularly myricetin, quercetin and kaempferol. In silico studies of these flavonoids within the active site of gastric proton pump showing pronounced inhibition further ascertained the antiulcer potential. Thus, it can be concluded that *Heliotropium crispum* roots can act as a potent gastro-protective agent, whereas further preclinical studies should be performed to further consolidate the obtained results. However, it is highly recommended to explore the molecular pathway involved in the reduction of ethanol induced ulcer in rats 

## Figures and Tables

**Table 1:** Fourier-transform infrared spectroscopy (FTIR) peak values (cm^−1^) and functional groups interpretation of *H. crispum* plant powder.

Peak Value	Functional Group	Functional Group Name	Vibrations
3296.5	Ac-O-H	Carboxylic acids	
	N-H	Primary amides	
	R-C=O-NR_2_	Secondary amides	
	O-H	Polymer alcohols and phenols	
2918.0	H-OH	Hydroxyl group	
	-NH	Secondary free or bonded NH strand	
1596.3	(i).Secondary NH bond	Secondary aromatic amine	Deformed bond
	(ii).N=N	Azo compound	
	(iii).C=N	Imine compounds	Bent bond
	(iv).R-NH_2_	Primary aliphatic amine	Deformed
	(v).RC(=O)NR/R//	Primary amide	Deformed or bent bond
1410.0	R_2_-CHOH	Tertiary alcohols	Deformed form
1241.8	R-C_6_H_5_-R	Alkyl aryl group	
	2R-C_6_H_5_-	Diaryl group	
1013.5	C-F	Floro group(halo alkane)	
	C-OH	Primary alcohol	
568.9	C-Br	Bromo group (halo alkane)	
490.8	C-I	Iodo group (halo alkane)	

**Table 2:** HPLC profile of *H. crispum* total methanol extract (CME) as well as its various fractions.

Fraction	Retention Time	Compound
CME	4.933	Myricetin
Chloroform	5.240	Myricetin
	8.862	Quercetin
Ethyl acetate	5.433	Myricetin
	11.381	Kampherol
*n*-Butanol	5.387	Myricetin
Remaining Aqueous	No peaks found	No compound detected

**Table 3:** Antiulcer effect of *H. crispum* CME and different fractions on different gastric parameters.

Groups	Ulcer Number	Incidence of Ulcer	Ulcer Score	Ulcer Index	% Inhibition
Normal	0 ± 0 ^a^	0 ^a^	0 ± 0 ^a^	0 ^a^	0 ^a^
Ethanol	11.33 ± 1.909	100	13.833 ± 2.27 ^a^	125.17 ^a^	0 ^a^
Omeprazole	0.167 ± 0.167 ^a^	16.667 ^a^	0.166 ± 0.17 ^a^	17 ^a^	84.410 ^a^
CME	0.167 ± 0.167 ^a^	16.667 ^a^	0.500 ± 0.22 ^a^	17.33 ^a^	86.150 ^a^
*n*-Hexane	0.833 ± 0.307 ^a^	66.67 ^a^	10.667 ± 0.667 ^a^	69.33 ^a^	44.600 ^a^
Chloroform	0.167 ± 0.167 ^a^	16.667 ^a^	0.1667 ± 0.1667 ^a^	17 ^a^	86.418 ^a^
Ethyl acetate	0.170 ± 0.166 ^a^	16.667 ^a^	0.167 ± 0.166 ^a^	17 ^a^	86.418 ^a^
*n*-Butanol	1.500 ± 0.340 ^a^	83.333 ^a^	1.500 ± 0.223 ^a^	86.17 ^a^	31.025 ^a^
Remaining aqueous	0.830 ± 0.307 ^a^	66.667 ^a^	1.000 ± 0.258 ^a^	68.5 ^a^	45.272 ^a^

^a^ *p* < 0.01 versus the standard group.

**Table 4:** Effect of *H. crispum* CME and different fractions on gastric mucus content and gastric protein content.

Groups	Total Protein Content	Gastric Mucin Content
Normal	258.42 ± 6.65 ^c^	507.83 ± 8.36 ^c^
Ethanol	89.33 ± 7.43 ^c^	428.67 ± 9.12
Omeprazole	217.00 ± 15.24 ^c^	481.83 ± 4.79 ^b^
CME	248.50 ±13.63 ^c^	481.83 ± 14.15 ^b^
*n*-Hexane	160.00 ± 10.88 ^c^	444.17 ± 15.60 ^ns^
Chloroform	252.33 ± 3.56 ^c^	498.67 ± 7.83 ^c^
Ethyl acetate	234.83 ± 7.93 ^c^	460.00 ± 13.05 ^ns^
*n*-Butanol	198.50 ± 5.36 ^c^	503.50 ± 4.09 ^a^
Remaining aqueous	184.50 ± 5.50 ^c^	480.33 ± 12.18 ^c^

^a^ *p* < 0.5, ^b^ *p* < 0.1, ^c^ *p* < 0.01 versus the standard group; ns: non-significant.

## Data Availability

Data are available in the manuscript.

## Supplementary Materials

The following supporting information can be downloaded at: https://www.mdpi.com/article/10.3390/metabo12080750/s1. Figure S1: FTIR spectrum of *Heliotropium crispum* aerial parts powder; Figure S2: HPLC chromatogram of the different extracts of *Heliotropium crispum* aerial parts.
