# Supplementary material for: Metabolic Profiling of Heliotropium crispum Aerial Parts Using HPLC and FTIR and In Vivo Evaluation of Its Anti-Ulcer Activity Using an Ethanol Induced Acute Gastric Ulcer Model
Source: Metabolites. 2022 Aug 16;12(8):750. doi: 10.3390/metabo12080750 (PMC9416604; doi:10.3390/metabo12080750)

Metabolic Profiling of *Heliotropium crispum* Aerial Parts Using HPLC and FTIR and *In Vivo* Evaluation of its Anti-Ulcer Activity Using Ethanol Induced Acute Gastric Ulcer Model

Supplementary data

Figure S1: FTIR spectrum of *Heliotropium crispum* aerial parts powder

Figure S2: HPLC chromatogram of the different extracts of *Heliotropium crispum* aerial parts

Figure S1: FTIR spectrum of *Heliotropium crispum* aerial parts powder

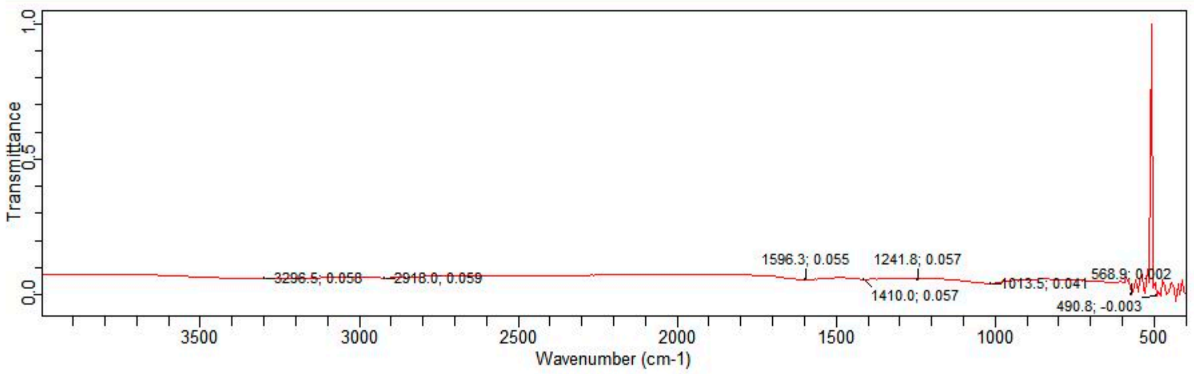

Figure S2: HPLC chromatograms of the different extracts of *Heliotropium crispum* aerial parts

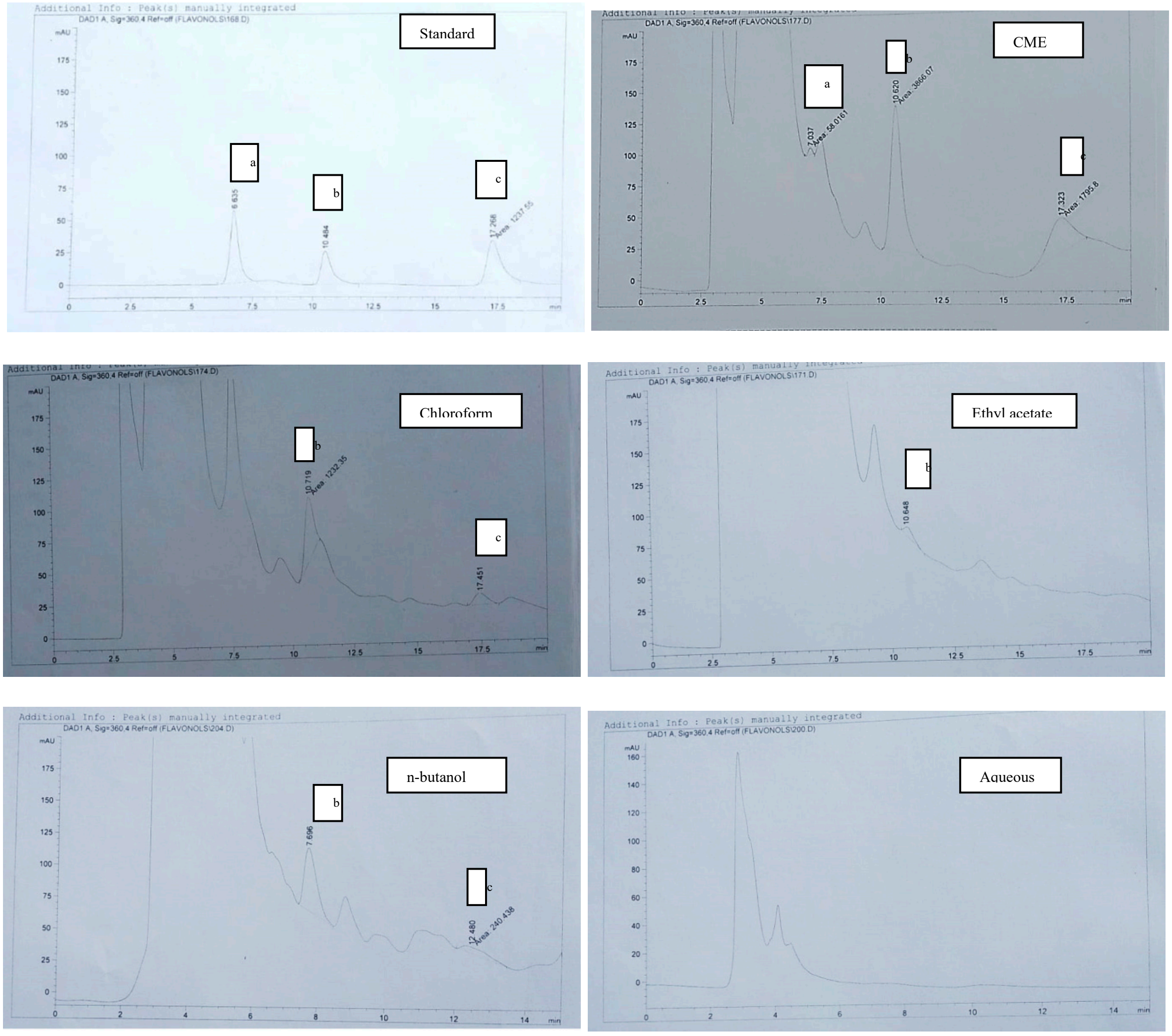

Supplement: Supplementary file 1 [file metabolites-12-00750-s001.zip › metabolites-1830864-supplementary.pdf]
